# Appropriate Foods

**Published:** 1883-04

**Authors:** 


### APPROPRIATE FOODS.

IF we examine the teeth of a dog we see that they are long and pointed, and that they are perfectly adapted for destroying other animals, and for tearing their flesh into pieces small enough for him to swallow; but that they are not constructed so that he could masticate his food. The only conclusion we can draw from this fact is that nature intended that the dog should live mainly on the flesh of other animals. We find that the horse possesses two kinds of teeth: the cutting teeth in front, and back of these, a powerful set of grinding teeth; but he has no pointed, or canine teeth. It is apparent then, that the cutting teeth were intended to bite off grass or other vegetable foods, to be passed back to the grinders and there prepare for the stomach. The horse's food, therefore, must be entirely vegetable.

But man possesses the three varieties of teeth; the cutting or front teeth, then the four canine teeth, and back of these the grinding teeth. The Power which created all things, created them for a purpose. Nature would never have provided us with canine teeth if she had intended that we shonld live exclusively on a vegetable diet; and although it may be possible for men to run counter to the plain indication of nature, and use a vegetable diet exclusively for years, there are penalties attached to the violation of these laws, just as there are to all unnatural practices, and sooner or later the penalty follows. The doctrine of the vegetarians is not only absurd but evil. We are acquainted with consistent, practical vegetarians; but not one who is not prematurely old, and not one who is not the victim of disease.

"Variety is the spice of life." That man is wisest, healthiest and happiest who partakes moderately of the bounties nature spreads before him; of fish, fowl, flesh, and of the infinite variety of wholesome foods furnished by the vegetable kingdom. ALL these things are good for us if used intelligently; and any man who has the wisdom to properly regulate his appetite need never be a vegetarian.

We are allowed a wide discretion as to the uses we may make of the products of the earth. It could never have been intended to restrict us exclusively to either a vegetable or an animal diet; but that we should partake of both under the guidance of reason and experience to restrain us from excesses.

